# Verifying monitor unit calculations for tangential breast fields

**DOI:** 10.1120/jacmp.v7i2.2177

**Published:** 2006-05-25

**Authors:** Ian Kay, Peter Dunscombe

**Affiliations:** ^1^ Tom Baker Cancer Centre 1331 29th Street NW Calgary Alberta T2N 4N2; ^2^ Departments of Oncology and Physics and Astronomy University of Calgary 2500 University Drive NW Calgary Alberta T2N 1N4 Canada

**Keywords:** radiotherapy, breast, tangent field, treatment planning, quality assurance

## Abstract

An essential component of quality assurance in radiation therapy is verifying the accuracy of monitor unit calculations. Differences between sophisticated algorithms using 2.5D or 3D calculations and simpler monitor unit check algorithms or hand calculations assuming a flat water phantom must be expected. For many anatomical sites, such differences are small and of little or no consequence in the context of expected clinical impact. However, for tangential breast fields the discrepancies are considerably larger than those that would generally be considered acceptable. A simple model to reconcile the differences between sophisticated and simple algorithms is presented, based on replacing the breast contour with a triangular or elliptical contour and using this to estimate an equivalent rectangular prism providing equivalent scatter to the prescription point. The elliptical approximation reconciles the observed differences in calculated monitor units. The analysis we present can assist the treatment planning physicist in selecting a method and tolerance window for verifying monitor unit calculations for tangential breast fields.

PACS: 87.53.Kn, 87.53.Tf, 87.53.Xd

## I. INTRODUCTION

External beam therapy quality assurance (QA) requires that plans be validated,^(1)^ and an independent monitor unit calculation is an essential part of that QA process. At the Tom Baker Cancer Centre our policy in this regard requires all plans (generated by ADAC Pinnacle™) to be downloaded to RadCalc™ for the independent monitor unit check. If disagreement between Pinnacle™ and RadCalc™ calculated monitor units is larger than the greater of 2% or 2 monitor units (MU) for any field, the plan must be referred to a physicist for final approval. This policy provides adequate assurance of the accuracy of the calculated monitor units while limiting the workload on the physicists. As will be seen below, fewer than 25% of beams fail to meet this established criterion for all anatomical sites except the breast, where no beams display agreement between Pinnacle™ and RadCalc™ of 2% or better.

Missing tissue, field “flash,” and an oblique irradiation of the surface combine to make the dosimetry of breast irradiation using tangent fields complex. Modern treatment‐planning systems (such as Pinnacle™) meet this challenge by accounting for the external body surface (hence field “flash” and missing tissue) and oblique incidence inherently in their algorithm.

Validation of the monitor unit calculation is performed either using hand calculations or one of many software packages, both “home‐grown”^(2)^ and commercial. These methods usually either assume or use data measured for radiation beams perpendicular to a flat water surface and with full scatter conditions. These conditions are not realized in tangential breast fields; hence, discrepancies arise when comparing the monitor unit calculations.

Due to the approximations in monitor check routines, strategies for validating breast plans have been developed. Many of these involve some adjustment of the field size to an “equivalent square” field and are frequently based on experience. For example, Ayyangar et al.^(3)^ describe a method of correcting the “second check” for the effects of flash and missing tissue. This method requires a measurement of a “missing tissue factor” as a function of a “percent missing tissue index,” and an estimated equivalent square based on the beam's‐eye‐view extent of field flash. They report 1.5% agreement of monitor units over 15 fields examined, with a standard deviation of 2.0%.

We have investigated alternative schemes for correcting monitor units calculated for tangential breast fields under simple geometrical conditions. The schemes are based on the breast profile and the location of the prescription point. The notion of equivalent square is extended, replacing the breast contour with an “equivalent rectangular parallelepiped,” which would provide similar scatter to the prescription point. Performance of this method appears similar to that of Ayyangar et al.^(3)^

Currently, our clinic plans in 2.5D using a single digitized breast profile (in Pinnacle™) and checked either with software (RadCalc™) or a hand calculation, resulting in the disagreements described here. We anticipate that full 3D planning will make reconciliation of the monitor unit calculations more difficult. This initial study showed that an equivalent scattering area approximation works well in 2.5D, and extension to an equivalent scattering volume in 3D is a promising strategy for using current second check tools and reconciling the differences observed.

## II. METHODS

**Figure 1:**
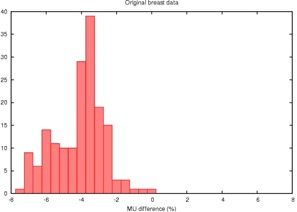
Histogram of the percent disagreement in monitor units calculated by RadCalc™ compared to Pinnacle™ for 86 tangential breast fields showing an average difference of 4% due to differences in the computational algorithm. MU=monitor units

Eighty‐six tangential breast plans, each with a medial and a lateral field, and each treated at 6 MV, were culled at random from our clinical database. These 2.5D plans were prepared by acquiring a 2D contour of the breast and using ADAC Pinnacle™ to calculate both the dose distribution and the monitor units for each field. According to our planning protocol, 2 cm of flash is added to each field, and the dose is prescribed to a point approximately midway between the beam entry points and one‐third of the distance from the posterior field border to the anterior breast surface (Fig. 1). The fields included in this study were asymmetric, with wedges and half‐blocking in most cases, although in some cases 1 cm or 2 cm are allowed on the chest wall side of the central axis.

The resulting plans were exported to RadCalc™ for a second check. Comparing monitor units from Pinnacle™ and RadCalc™, we observe a difference of −4.4% and a standard deviation of 1.4%. (RadCalc™ prescribed fewer monitor units than ADAC Pinnacle™.)

As a point of comparison, 405 fields treating sites other than breast/chest wall were examined. The mean difference between the monitor units calculated by RadCalc™ and Pinnacle™ is −0.7%, with a standard deviation of 1.6%, consistent with the work of Leszczynski and Dunscombe.^(4)^ The largest deviations (~8%) occurred for fields treating the arm, axilla, or sinus, where contour, flash, and inhomogeneity are expected to produce discrepancies. It should be noted that these other fields are almost all full 3D conformal plans and usually included corrections for tissue heterogeneity, whereas our breast tangent fields are usually calculated assuming homogeneous tissue density. Yet for these other 405 fields, agreement is much better than for the breast fields, indicating that it is this particular geometry that is pushing RadCalc™ beyond the valid limits of its assumptions.

**Figure 2:**
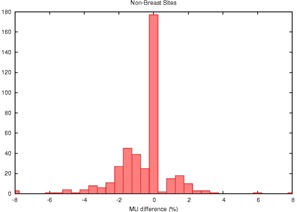
Histogram of the percent disagreement in monitor units calculated by RadCalc™ compared to Pinnacle™ for 405 fields applied to sites other than the breast. On average, the agreement is better and the distribution is tighter, demonstrating that tangential breast fields pose a special challenge. MU=monitor units

Plots of the Pinnacle™ versus RadCalc™ monitor units are highly linear and unrevealing; $R^{2}$ is in excess of 0.999 for both the breast and nonbreast data. The slope of the regression line is about 1.04 for the breast data versus 1.007 for the nonbreast site. More revealing are the histograms of the percentage disagreement of RadCalc™'s monitor units from that of Pinnacle™'s (Figs. 1 and 2).

The first and easiest correction to the geometrically simple approach (RadCalc™) is to reduce the field size for calculating phantom scatter by the amount of flash that we will denote by *f*. We have simply assumed that planners have rigorously held to the prescribed 2 cm of flash.

**Figure 3:**
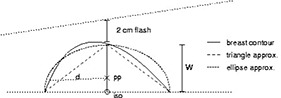
A typical breast contour (solid) can be approximated as a triangle (long dashes) or as an ellipse (short dashes). A half‐blocked tangential field is indicated incident from the left side. pp=prescription point;iso=isocenter

**Figure 4:**
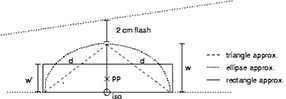
Replacing the triangle or ellipse in Fig. 3 with a rectangle of “equivalent mass.” pp=prescription point;iso=isocenter

We next need to account for an overestimation of tissue providing scatter to the prescription point. Consider an audacious assumption, that the amount of scatter to the prescription point would remain constant if the breast tissue were rearranged into a rectangular prism. The corrections we investigated are based on either a triangular or an elliptical approximation of the breast contour as shown in Fig. 3. The degree to which either approximates the breast contour will clearly vary for any given patient and field. Also note that we use the depth, *d*, to the prescription point as half of one dimension of the rectangle as shown in Fig. 4.

The rectangle width w’ is chosen such that the area *w*’ *d* is equal to the area of the triangle or the half ellipse in Fig. 3. The difference between *d* and the depth to the isocenter is ignored. This “equivalent rectangle” would be similar to the field illuminated by a beam of width $w^{'}$ on a flat water phantom, ignoring the divergence of the field's upper border. For our two approximations:

(1) $$w^{\prime }(2d)=2\;{(}^{1}{/}_{2}\;wd)\;\text{or}\;w^{\prime }=w/2\;(\text{triangle});$$

(2) $$w^{\prime }(2d)=\pi /2\;wd\;\text{or}\;w^{\prime }=\pi w/4\;(\text{ellipse}).$$

The original rectangular field of length *L* and width w+f had an equivalent square field size F=2L(w+f)/(L+w+f). The equivalent rectangular scatterer has a width $w^{'}$ as given above. The equivalent square field size *F* is then replaced by $F^{'}$, calculated using the original field length *L*, but the revised field width $w^{'}$ so that $F^{'}=2{\text{Lw}}^{'}/(L+w^{'}$); hence

(3) $$F^{\prime }/F=(w^{\prime }/(w+f))(L+w+f)(L+w^{\prime }).$$

When calculating our second check (using RadCalc™, hand calculation, or other based on full scatter conditions), we replace the (w+f) by *L* field of equivalent square size *F*, with a new field $w^{'}$ by *L* and equivalent square size $F^{'}$. This change of field size should account for most of the missing scatter, by changing the values of $S_{p}$ and tissue phantom ratio (TPR) used in the calculation. This can be carried out with a simple multiplicative correction factor:

(4) $$S_{p}(F)\text{TPR}(F,d)/S_{p}(F^{\prime })\text{TPR}(F^{\prime },d),$$

with $F^{'}$ related to $F^{'}$ as given above. This factor is greater than 1, and dividing the number of monitor units calculated for a flat water phantom by this factor should approximate the correct number of monitor units (as given by Pinnacle™ or other modern planning systems) within the geometrical assumptions of the triangle and ellipse.

## III. RESULTS

**Figure 5:**
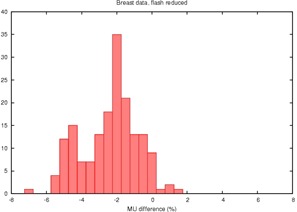
Histogram of the percent disagreement in monitor units after RadCalc™'s field dimension is altered to account only for excess field flash

Monitor units were recalculated in RadCalc™ for our selected 86 pairs of tangential breast fields, reducing the field width by 2 cm to correct for field flash. This leaves the effect of surface contour uncorrected. The results are summarized in the histogram in Fig. 5 and in Table 1.

**Table 1:** Performance of correction strategies

	No correction	Flash reduction only	Triangle correction	Ellipse correction
average difference	−4.4%	−2.7%	2.7%	−1.0%
standard deviation of difference	1.4%	1.7%	1.9%	1.6%

**Figure 6:**
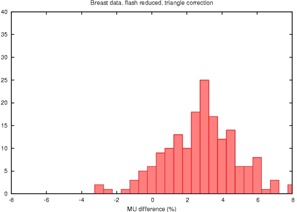
Histogram of the percent disagreement in monitor units after RadCalc™'s field dimension is altered to account both for excess field flash and a triangular estimate of the missing tissue

**Figure 7:**
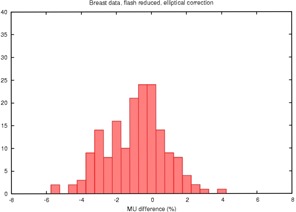
Histogram of the percent disagreement in monitor units after RadCalc™'s field dimension is altered to account both for excess field flash and an elliptical estimate of the missing tissue

The proposed correction factor in Eq. (4) was then applied, using both the triangle and elliptical approximations, yielding the results in Table 1 and the histograms in Figs. 6 and 7.

The sample data have a range of depths to the prescription point of 9.1±1.6cm and field widths of 10.2±3.1cm. (Results are quoted with 1 SD, corresponding to 68% CI.)

As a further simplification, consider approximating the superior/inferior length of the treatment field to be a constant nominal length of 20 cm (our data have an average of 19.0±2.2cm). This alters the data only slightly to that shown in Table 2.

**Table 2:** Performance of correction strategies with the further assumption of a constant 20 cm field length

	No correction	Triangle correction	Ellipse correction
average difference	−4.4%	2.6%	−1.2%
standard deviation of difference	1.4%	2.0%	1.7%

## IV. DISCUSSION

Flash reduction alone provides a partial reconciliation of the monitor unit disagreement, but leaves an average disagreement of 2.7% unresolved.

From Table 1 and Fig. 6, it appears that the triangle approximation overcorrects the monitor units in most cases. This is expected because it likely overestimates the amount of missing tissue as can be appreciated in Figs. 2 and 3. The increase in standard deviation also indicates that this approximation is not performing well. In contrast, the elliptical approximation performs quite well.

Assuming a constant field length as in Table 2 preserves the same pattern; the triangle approximation overcorrects, and the ellipse appears to perform well. Making this further simplifying assumption allows us to produce a 2D table of correction factors that are functions of only field size and depth.

The elliptical approximation is surprisingly good at correcting the monitor unit calculation, better than we expected given the very crude approximations being made. The range of differences (max to min) in the original uncorrected data is 0.0% to −7.7%. After correction with the elliptical model the range is 3.4% to −4.2%. The mean is being corrected for lack of scatter, contour, etc., while preserving the range of values, increasing our faith in the results.

TG‐40^(1)^ recommends that primary and secondary monitor unit calculations should agree to within ±2%. The data in Table 1 suggest that uncorrected tangential breast fields would meet this criterion only 4.3% of the time. Using the elliptical correction factor, even with the residual −1.2% difference, 68% of the plans would pass this test. As a point of comparison, for the 405 fields delivered to sites other than breast 75% would meet the 2% tolerance level, and 25% would require checking by a physicist.

However, the question does remain as to whether or not one needs to apply a correction factor to check monitor units for tangential breast fields. If the analysis above, together with the experimental validation of Pinnacle™ by Ayyangar et al.,^(3)^ can be used to infer both that the physical reason for the observed discrepancies is understood and that Pinnacle™ (or any other modern algorithm) is inherently accurate under the relevant geometric conditions, then it would be sufficient to specify that secondary monitor units be in the range of −6.4% to −2.4% to pass the checking procedure, encompassing 80% of the breast plans. Implementation of such a policy provides the same assurance of accuracy as for other anatomical sites and results in a site‐independent workload for physicists performing plan checks.

If one is confident that the increase of standard deviation from 1.4% to 1.7% in Table 2 was due to variation in flash and field length, and not related to any serious errors in planning, one might increase the acceptance criteria to 1.7/1.4×2%=2.4% or 1.4 standard deviations. This would pass about 84% of plans, and 16% would still be checked. Or one could select a range that conforms to one's comfort level based on an estimation of the frequency of a serious error. The approach presented here allows each center to make an informed decision.

## V. CONCLUSIONS

We have considered the problem of disagreement in monitor unit calculations between a primary and a second check for 2.5D planning of tangent breast fields. The disagreement arises when the second check is performed with a simplified algorithm that does not account for the absence of full scatter conditions.

We have described a simplified method of correcting for the absence of full scatter conditions in the case of tangential breast fields based on a geometrical estimate of an equivalent scattering rectangular parallelepiped. The assumptions are justified on physical grounds and supported by the statistics demonstrating the efficacy of the method. The results presented give the clinical physicist the option of correcting monitor units for better agreement with more accurate 2.5D calculations or simply shifting the tolerance window to accommodate the approximations made during checking. The analysis can, and should, be performed by any user by a retrospective examination of plans to ensure that they get acceptable performance before implementing it as a solution in their clinic.

Extension of this method to the case of full 3D planning is obvious in principle; an estimate of the treated volume of breast tissue could be used to calculate an equivalent rectangular parallelepiped. Extension of our ellipse to a half ellipsoid is tempting, but may prove to be a poor approximation to real anatomy. As our clinic moves to 3D breast planning, we hope to collect the data that would allow us to test extensions to this simple model.

## ACKNOWLEDGMENT

Thanks to Mr. S. Morgan for his assistance collating and analyzing this data.
